# Branch retinal artery occlusion secondary to high-altitude exposure and diabetic retinopathy: a case report

**DOI:** 10.1186/s12886-020-01563-1

**Published:** 2020-07-11

**Authors:** Xue Feng, Luping Wang, Haiwei Wang, Hong Qi, Jianqiang Zhang, Yanling Wang

**Affiliations:** 1 Department of Ophthalmology, Beijing Friendship Hospital, Capital Medical University, NO.95 Yong’an Road, Xicheng District, Beijing, 100050 China; 2 Department of Ophthalmology, Beijing Moslem People’s Hospital, Beijing, China; 3 Department of Ophthalmology, Fuxing Hospital, Capital Medical University, Beijing, China; 4 Department of Ophthalmology, Peking University Third Hospital, Beijing, China

**Keywords:** Branch retinal artery occlusion, High altitude, Diabetic retinopathy, Hyperbaric oxygen

## Abstract

**Background:**

To report a case of branch retinal artery occlusion (BRAO) secondary to high-altitude exposure and diabetic retinopathy (DR), and to characterize the retinal changes before and after hyperbaric oxygen (HBO) treatment.

**Case presentation:**

We present a case of a 42-year-old man with DR who travelled to Tibet (in China, 3800 m/12467 ft. above mean sea level). The day after the end of his journey, the patient presented with acute, painless visual loss and visual field loss in his left eye. He was then diagnosed with BRAO, which is an acute blockage of blood flow. After HBO treatment, visual acuity and visual field were improved.

**Conclusions:**

High-altitude exposure and DR may be considered as relevant risk factors for BRAO. The ophthalmologist should be aware that the BRAO might be a rare presenting sign of high-altitude retinopathy (HAR), particularly in patients with DR. HBO treatment can be considered as a choice for ophthalmologists on treatment against BRAO.

## Background

Travelling to altitudes of 2500 m or more may put people at risk of high-altitude retinopathy (HAR), which is an acquired vascular retinopathy characterized by dilated veins and retinal hemorrhages [1]. Usually, vision is not affected by HAR unless it involves the macula [2]. HAR tends to resolve spontaneously, and in most cases, treatment is not required; nevertheless, systemic altitude illness needs to be treated. The mechanism of HAR remains unknown [3]. It has been hypothesized that decreased arterial oxygen results in vascular incompetence [4]; however, it remains unclear what happens to the retina of patients with diabetic retinopathy (DR) exposed to high altitude. Branch retinal artery occlusion (BRAO) secondary to high-altitude exposure is uncommon in clinical practice, particularly in patients with DR. Herein, we reported on a peculiar case of BRAO involving concurrent high-altitude exposure and DR without other causative agents. In addition, we summarized the characteristic features of the changes in the retina before and after hyperbaric oxygen (HBO) treatment.

## Case presentation

A 42-year-old man (lowlander, Beijing, China, 43.5 m/142.7 ft. above mean sea level) presented with acute, painless visual loss and visual field loss in his left eye. The patient travelled to Tibet before the onset of symptoms when he took 1 day to ascend to the high altitude by car. He spent 1 week at high altitude before the descent back, which also took him 1 day by car. The complaints began at a low altitude after the end of his journey. The patient had type II diabetes, which was controlled by insulin for 10 years. He also used metformin for some time. The patient underwent binocular subtotal panretinal photocoagulation for the treatment of DR at 3 months before his journey. The patient did not have any other ocular treatments, such as intravitreal injections or intraocular surgery. According to the severity of DR from the description of the patient, the left eye was slightly worse than the right eye. Blood glucose was not monitored during the high-altitude journey. The patient had no history of smoking, hypertension, and hypercholesterolemia. Multiple carotid atherosclerotic plaques were shown by the Doppler examination. Increased blood cell counts for white blood cell (WBC), red blood cell (RBC), hemoglobin (HB) and packed cell volume (PCV) were revealed by hematologic examination. Decreased prothrombin time (PT) and increased prothrombin time activity were revealed by hematologic examination. All of the systemic examination parameters are shown in Table 1.

**Table 1:** Systemic examination parameters of the patient

Characteristic	Measured value	Reference values
Blood pressure (mmHg)	135/85	< 140/90
Pulse rate (/min)	88	60–100
Axillary body temperature (°C)	36.2	36–37
FBG (mmol/L)	6.78^a^	3.9–6.1
HbA1c (%)	6.4^a^	4.0–6.0
WBC (10^9^/L)	12.93^a^	3.5–9.5
RBC (10^12^/L)	6.23^a^	4.3–5.8
HB(g/L)	180^a^	130–175
PLT (10^9^/L)	276	125–350
PCV (%)	52.4^a^	40–50
CRP (mg/L)	0.5	0.0–8.0
PT(S)	10.4^a^	11.0–14.0
PT% (%)	127^a^	66–110

*FGB* Fasting blood glucose, *WBC* White blood cell, *RBC* Red blood cell, *HB* Hemoglobin, PLT platelet, *PCV* Packed cell volume, *CRP* C-reactive protein, *PT* Prothrombin time. ^a^ measured values are out of reference values.

**Fig. 1:**
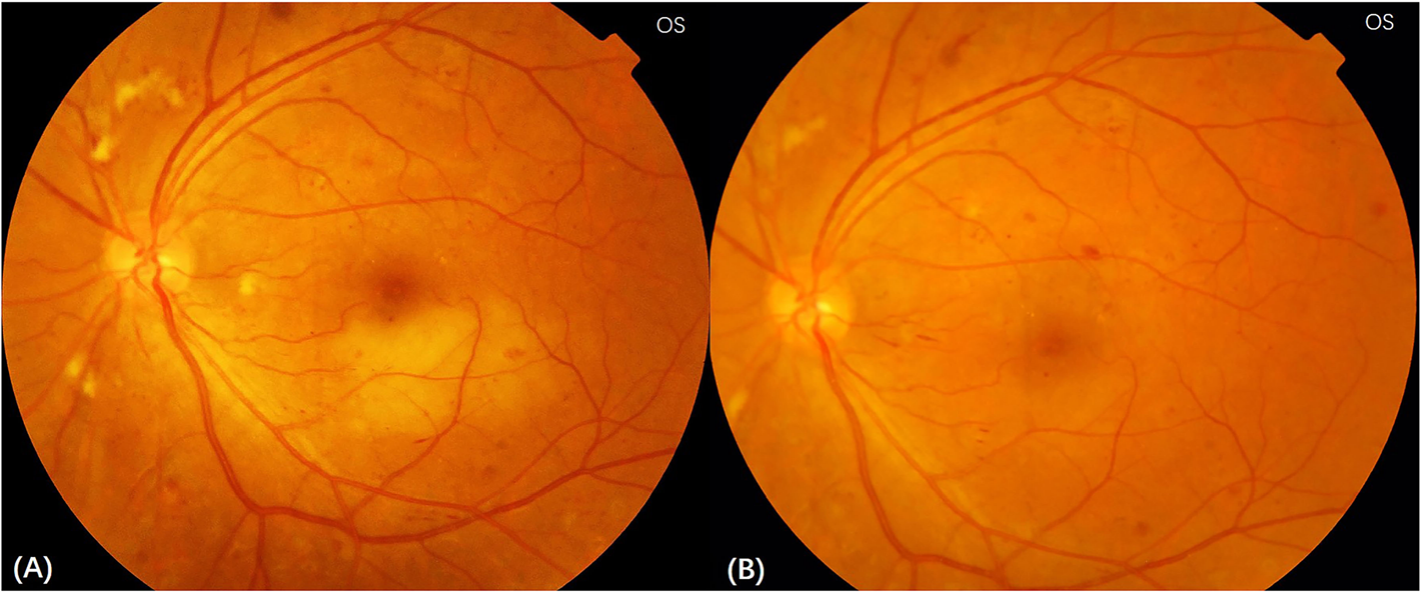
**a** Color fundus photograph of the left eye showing an area of superficial retinal whitening inferior to the fovea along the distribution of the inferotemporal branch retinal artery before the HBO treatment. **b** Color fundus photograph of the left eye showing superficial retina whitening disappeared after the HBO treatment

**Fig. 2:**
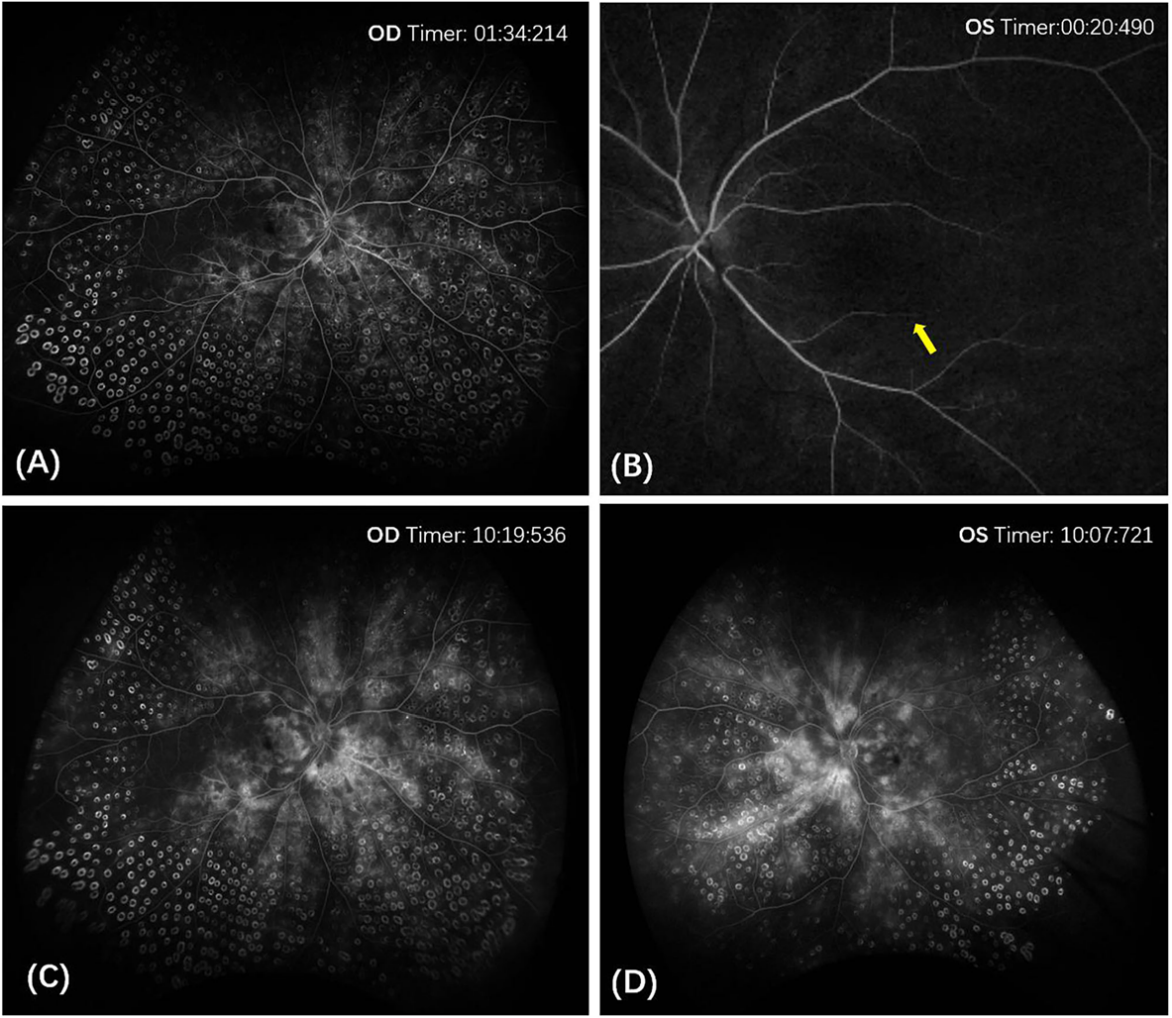
(**a**, right eye; **b**, left eye) Ultra-widefield fundus fluorescein photographs, the yellow arrow in image B indicating delayed arterial filling corresponding to the area of retinal edema in the early phase. (C, right eye; D, left eye) Ultra-widefield fundus fluorescein photographs showing capillary nonperfusion around the optic disc and leakage at the posterior pole in the late phase

**Fig. 3:**
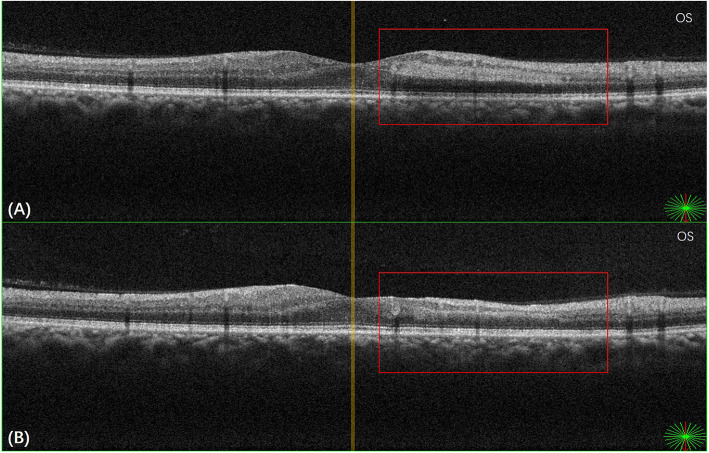
**a** OCT scan of the left eye showing hyper-reflective band in the inner plexiform and inner nuclear layers and thickening of the retinal layers before the HBO treatment. **b** OCT scan of the left eye showing the narrowing of hyper-reflective band and the thinning of the retinal layers after the HBO treatment

**Fig. 4:**
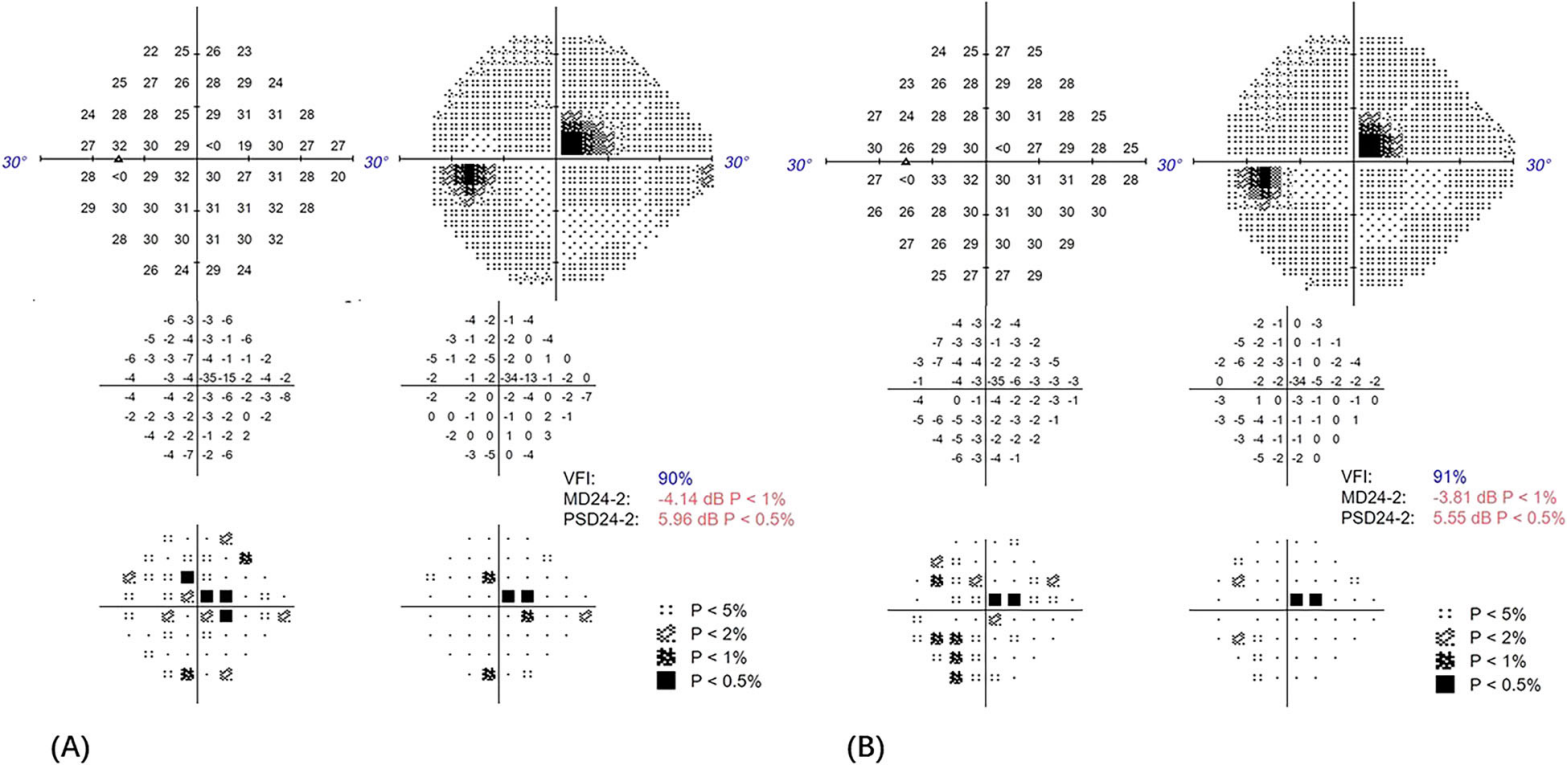
**a** Humphrey visual field showing central scotomas corresponding to the area of BRAO before the HBO treatment in the left eye. VFI,90%; MD24–2, − 4.14 dB; PSD24–2, 5.96 dB. **b** Humphrey visual field showing central scotomas had become. smaller after the HBO treatment in the left eye. VFI,91%; MD24–2, − 3.81 dB; PSD24-. 2, 5.55 dB.

The best-corrected visual acuity (BCVA) in the right and the left eye were 20/25 and 20/40 (Snellen Chart), respectively. Non-contact intraocular pressure was 17 mmHg in the right eye and 18 mmHg in the left eye. Anterior segment examinations were normal in both eyes. Ophthalmoscopy revealed hemorrhages, cotton wool spots, and laser spots in both eyes. Superficial retinal whitening inferior to the fovea along the distribution of the inferotemporal branch retinal artery was revealed by color fundus photograph of the left eye (Fig. 1). Delayed arterial filling corresponding to the area of retinal edema in the early phase and capillary nonperfusion around the optic disc, leakage at the posterior pole in the late phase were revealed by ultra-widefield fluorescein angiography (UWFA) (Fig. 2). Hyper-reflective band in the inner plexiform and inner nuclear layers and thickening of the retinal layers were shown by spectral-domain optical coherence tomography (SD-OCT) (Fig. 3). Central scotomas corresponding to the area of BRAO in the left eye were shown in the Humphrey visual field (Fig. 4).

The HBO treatment, which included daily sessions lasting for 110 min at 2.0 absolute atmospheres, was performed for 10 days. The BCVA in the left eye on the fourth and sixth day of the HBO treatment were 20/33 and 20/25, respectively. On the first day after the end of the HBO treatment, the BCVA in the left eye was 20/20, and it remained unchanged at 1 month after the HBO treatment. Superficial retina whitening of the left eye disappeared, which was revealed by color fundus photograph (Fig. 1). The narrowing of the hyper-reflective band and the thinning of the retinal layers were shown in SD-OCT (Fig. 3). Central scotomas of the Humphrey visual field had become smaller in the left eye (Fig. 4).

## Discussion and conclusions

Currently, there are only a few reports on retinal artery occlusion secondary to high-altitude exposure. A case of central retinal artery occlusion secondary to bilateral buried optic nerve drusen at high altitude was reported in 1995 [5], while another case of central retinal artery occlusion caused by the expansion of intraocular gas during mountain travel at high altitude was reported in 2002 [6]. A recent report has shown that cilioretinal artery occlusion and related central retinal vein occlusion occurred as a complication following high-altitude exposure [7]. It has been speculated that the reason for a few reports of retinal artery occlusion secondary to high-altitude exposure might be that some clinicians do not master the pathogeny of retinal vascular occlusion, thus do not inquire the patient’s travel history or consider high altitude as a risk factor. Possible reasons for retinal artery occlusion secondary to high-altitude exposure may be related to hematocrit, hemoglobin concentration, and blood viscosity that were all increased in the hematologic examination of high-altitude climbers, which indicated the higher coagulative activity, as the present case shown [1]. The patient was in a state of hypercoagulability. The atmospheric pressure decreases along with the increase of the altitude, after which the retinal arteries and veins tend to dilate. Retinal vascular occlusion in patients with circulatory impairment has been shown to be triggered by reactive vasoconstriction, which may occur during the descent [8]. Furthermore, hypoxia has an important role in the development of retinal artery occlusion. Hypobaric hypoxia caused thrombosis, which further decreased the oxygen transport capacity. The patient, in our case began to experience disturbances at low altitude after the end of his journey. It is possible that reactive vasoconstriction and hypoxia injury of capillary endothelium may be the cause of the delayed vasculopathy [9].

Furthermore, there are few reports on cases with BRAO secondary to DR even in the absence of high-altitude exposure. Arterial attenuation, which is an important sign of retinal artery occlusion, is widely present in patients with DR, particularly in patients with panretinal photocoagulation [10]. Accordingly, it is difficult to detect retinal artery occlusion in patients with DR. As shown in our case, characteristics of BRAO were atypical in fluorescein angiography. The arterial lumen in patients with DR was less elastic and smaller, thus making emboli easier to be trapped [10]. Consequently, retinal artery occlusion is more likely to happen. On the other hand, patients with diabetes are more likely to have thrombosis [11]. In addition, patients with diabetes may suffer from the underlying ocular ischemic syndrome. In the present case, multiple carotid atherosclerotic plaques were shown by the Doppler examination. It is possible that when the central artery perfusion pressure is low, and without any emboli, the blood vessels tend to close more easily [12]. In addition, local choroidal circulation was damaged by previous panretinal photocoagulation [13]. The development of ocular ischemic syndrome may be aggravated by choroidal ischemia, which cannot be detected by fluorescein angiogram or by carotid artery doppler.

What happens to the retina of patients with DR exposed to high altitude? Both DR and high-altitude exposure are risk factors of BRAO, so there could be a synergistic effect between the two conditions [14]. A previous study has shown that acute high-altitude exposure-related hypoxia leads to a slight increase in central retinal thickness [15]. Another research has reported that high-altitudes exposure may accelerate the development of DR in athletes [16]. The patient, in our case, had diabetes for 10 years. Subtotal panretinal photocoagulation was performed before his high-altitude journey to Tibet. Blood glucose was not monitored during the journey. It is speculated that DR and high-altitude exposure had a synergistic effect on the occurrence of BRAO. The BRAO occurred in the left eye with relatively severe DR, not in the right eye with mild DR. Leal’s study [17] showed that in the development of DR, basal membrane thickening can contribute to vascular occlusion and retinal hypoxia. These conclusions need to be confirmed by further prospective research.

Previous studies have shown that HBO treatment is a safe, manageable, low-cost, and effective treatment for retinal artery occlusion [7]. HBO treatment have an effect on visual outcome in patients with BRAO [18]. HBO treatment causes an increased solubility of oxygen of the blood (from 0.3 up to 6 vol%) [19], which is important for the choroidal vasculature delivering more oxygen to the retinal [20]. Retinal circulation perfusion could be increased by HBO treatment. Intraocular pressure and scleral vein pressure could also be decreased by HBO treatment, and then thrombus could be moved to a further location [21].. A previous study showed that HBO treatment had been used for diabetic macular edema [14]. As our case shown, the subjective symptom such as visual acuity was improved after HBO treatment. Central scotomas of the visual field had become smaller after the HBO treatment, however learning also could contribute to the changes of the visual field. Objectively, the changes in fundus appearance and SD-OCT may reflect the natural history of the disease, not only the effects of HBO treatment. Prospective randomized controlled trials are needed to further confirm the actual improvement of HBO treatment. Long-term effects of HBO treatment on patients with BRAO need to be confirmed by follow-up.

In summary, high-altitude exposure and DR may be considered as relevant risk factors to BRAO. The ophthalmologist should be aware that the BRAO may be a rare presenting sign of the HAR, particularly in patients with DR. HBO treatment can be considered as a choice for ophthalmologists on treatment against BRAO.

## Data Availability

The datasets used and/or analyzed during the current study are available from the corresponding author on reasonable request.

## Abbreviations

BCVA: Best corrected visual acuity
BRAO: Branch retinal artery occlusion
CRP: C-reactive protein
DR: Diabetic retinopathy
FGB: Fasting blood glucose
HAR: High altitude retinopathy
HB: Hemoglobin
HBO: Hyperbaric oxygen
PCV: Packed cell volume
PLT: Platelet
PT: Prothrombin time
RBC: Red blood cell
SD-OCT: Spectral domain optical coherence tomography
UWFA: Ultra-widefield fluorescein angiography
WBC: White blood cell
